# Potential corner case cautions regarding publicly available implementations of the National Cancer Institute’s nonwear/wear classification algorithm for accelerometer data

**DOI:** 10.1371/journal.pone.0210006

**Published:** 2018-12-31

**Authors:** Hyatt E. Moore, K. Farish Haydel, Jorge A. Banda, Madalina Fiterau, Manisha Desai, Thomas N. Robinson

**Affiliations:** 1 Quantitative Science Unit, Stanford University, Stanford, CA, United States of America; 2 Stanford Solutions Science Lab, Division of General Pediatrics, Department of Pediatrics and Stanford Prevention Research Center, Stanford University, Stanford, CA, United States of America; 3 Department of Health and Kinesiology, Purdue University, West Lafayette, IN, United States of America; 4 Mobilize Center and Department of Computer Science, Stanford University, Stanford, CA, United States of America; Vanderbilt University, UNITED STATES

## Abstract

The National Cancer Institute’s (NCI) wear time classification algorithm uses a rule based on the occurrence of physical activity data *counts*—a cumulative measure of movement, influenced by both magnitude and duration of acceleration—to differentiate between when a physical activity monitoring (PAM) device (ActiGraph accelerometer) is being worn by a participant (wear) from when it is not (nonwear). It was applied to PAM data generated from the 2003–2004 National Health and Nutrition Examination Survey (NHANES 2003–2004). We discuss two corner case conditions that can produce unexpected, and perhaps unintended results when the algorithm is applied. We show, using simulated data of two special cases, how this algorithm classifies a 24-hour period with only 72 total counts as 100% wear in one case, and classifies a 24-hour period with 96,000 counts as 0.1% wear in another. The prevalence of like scenarios in the NHANES 2003–2004 PAM dataset is presented with corresponding summary statistics for varying degrees of the algorithm’s nonwear classification threshold (T). The number of participants with valid days, defined as 10 or more hours classified as wear time in a 24-hour day, increased while the mean counts-per-minute (CPM) decreased as the threshold for excluding non-wear was reduced from the allowed 4,000 counts in an hour. The number of participants with four or more valid days increased 2.29% (n = 113) and mean CPM dropped 2.45% (9.5 CPM) when adjusting the nonwear classification threshold to 50 counts an hour. Applying the most liberal criteria, only excluding hours as nonwear which contained 1 count or less, resulted in a 397 more participants (7.83% increase) and 26.5 fewer CPM (6.98% decrease) in NHANES 2003–2004 participants with four or more valid days. The algorithm should be used with caution due to the potential influence of these corner cases.

## Introduction

The increasing availability of wearable devices capable of continuously measuring accelerations at high rates for prolonged periods of time has provided researchers and consumers an excellent opportunity to objectively measure human physical activity. Physical activity monitoring (PAM) devices, such as those produced by ActiGraph (ActiGraph LLC, Pensacola, FL), often measure acceleration, in units of gravity, along one to three axes. These measures are commonly converted to and reported for each axis as *counts*; a unitless proxy that represents level of activity, obtained by filtering and summing the raw acceleration values over a fixed time interval known as an *epoch* through proprietary methods [1, 2]. Much work has gone into understanding these values as they relate to energy expenditure, and researchers often apply *cut-points* to categorize counts-per-minute (CPM) obtained from PAM devices into more general categories of activity such as sedentary behavior and light, moderate, and vigorous physical activity [3–13]. A challenge with PAM studies lies in identifying when a participant has removed his or her monitor, since the monitor will, under normal circumstances, continue to collect data regardless.

This poses difficulty in estimating quantities of interest related to physical activity and sedentary behavior. Thus, a number of classification algorithms have been developed for the purpose of distinguishing between periods of *wear* and *nonwear*, in order to improve accuracy of these activity estimates [4, 14–20].

This manuscript focuses on the nonwear classification algorithm developed by the National Cancer Institute (NCI) that we refer to as NCINW. NCINW was first implemented as part of a suite of SAS programs provided by NCI [21]. It is also included now with ActiGraph’s data analysis software ActiLife [22] and R’s *accelerometry* package (RAP) [15, 23]. We are specifically interested in the application of NCINW to examine uniaxial accelerometer data collected from youth and adults participating in the PAM portion of the 2003–2004 National Health and Nutrition Examination Survey (NHANES 2003–2004) [24–26]. These participants were instructed to wear an ActiGraph model 7164 uni-axial accelerometer (ActiGraph LLC, Pensacola, FL) set to measure vertical axis counts in 1-minute epochs, over their right hip, for 7 days. The dataset, consisting of 7,176 participants, is publicly available for analysis [27].

Troiano et al. were first to publish their findings on physical activity in the U.S. using NHANES 2003–2004 with NCINW. The authors rejected participant data deemed problematic (e.g. equipment returned was out of calibration) and focused on those with one or more valid days, which they defined as a 24-hour period with at least 10 hours of wear time classified by NCINW [4, 25]. Mean CPM and time spent in moderate or vigorous physical activity were reported for participants with ≥ 4 valid days, while estimates of adherence to minimum recommended physical activity levels (e.g. 30-min moderate-intensity activity for adults) were reported for participants with ≥ 1 valid days. Nonwear classification was thus a critical piece of the analysis. The authors explain, “nonwear is defined by an interval of at least 60 consecutive minutes of zero activity intensity counts, with allowance for 1–2 min of counts between 0 and 100” [4]. The source code clarifies that it is the number of consecutive minutes that occur within this *allowance* that is key in differentiating non-wear from wear (and not the proportion of the allowance per hour). Matthews *et al*, described the same classifier this way, “Periods of nonwear were defined as ending when count levels exceeded 100 counts/minute or when 3 consecutive minutes of observation were between 1 and 100 counts/minute.” [28] Interpreting this description with the source code, we see it is possible to classify a period of 60 minutes with 20 minutes of zero count activity and 40 minutes of 1 to 100 counts as nonwear.

An important question is how this may impact some of the findings based on analyzing the NHANES 2003–2004 with the NCINW. For example, if the algorithm misclassifies wear time as nonwear time, it may be that days that are valid for inclusion in an analysis by the author’s definition are incorrectly excluded from analysis and do not contribute to these findings. Matthews et al. [28], in a follow-up analysis, point out that the amount of wear time classified in NHANES 2003–2004 was “approximately 1.5 hours/day less than the average waking time reported in other national surveys”, and a limitation of their study. They speculate that these 1.5 hours/day differences likely reflect sedentary behavior that was misclassified and excluded as nonwear, in which case the study participants were even less active than shown. Finally, Choi et al found the NCINW more susceptible to classifying low-level, sedentary behavior as nonwear during an assessment of their own nonwear time classification algorithm [15]. Their efforts were aimed at improving the current state, and did so. However, the NCINW continues to be used today and it remains to be seen how its design may impact a study’s results.

We investigate the most extreme form of these corner cases using simulated data, and examine the implication of similar patterns on the analysis of NHANES 2003–2004. Our aim is to raise awareness of NCI’s nonwear classification algorithm’s (NCINW) behavior to certain patterns of activity as implemented, which may not be clear otherwise, and how this may impact PAM studies of free-living participants. We examine output from implementing NCINW through ActiLife and R in two scenarios that highlight potential classification problems. Our expectation is that there are conditions in which a large number of counts can accrue over a 24-hour day and still be considered nonwear, while a much smaller number of counts can accrue over a day and still be considered wear according to NCINW. We additionally examine NHANES 2003–2004 to determine the frequency with which these type of scenarios occur, and how the number of valid days, average CPM, and minutes spent in moderate intensity levels change when NCINW’s definition of wear is revised to include nonwear times with a minimum number of pre-classification counts.

## Methods

### Simulated data

To test our proposition, we created two 24-hour day scenarios, Scenario A and Scenario B, by repeating two hour-long patterns (Patterns A and B) of vertical-axis counts (1-min epoch), from midnight to 11:59PM (Tables 1 and 2). These scenarios were chosen to show the most extreme classification behavior of the NCINW in order to highlight problematic areas of the algorithm that may otherwise be missed. While fictitious, the hour-long activity described by Pattern A may be akin to the possibility of light physical activity, sedentary behavior or sleep being misclassified as nonwear, while the hour-long activity described by Pattern B shows similarities with possible low-level background noise during nonwear misclassified as wear. Pattern A begins with one minute of 0 counts, followed by two minutes of 100 counts each (i.e., 0, 100, 100). This three-minute pattern is replicated 20 times to create the hourly pattern that is then repeated 24 times to produce Scenario A. Pattern B begins with three minutes of 1 count each (i.e., 1,1,1), followed by 57 minutes of 0 counts. This hourly pattern is repeated for each hour of the day to produce Scenario B.

**Table 1:** Corner case hourly patterns. Pattern A consists of one minute of 0 counts followed by two consecutive minutes of 100 counts each. Pattern B consists of three minutes with 1 count each, followed by 57 consecutive minutes of 0 counts.

Minute	0	1	2	3	4	5	…	57	58	59
**Pattern A counts**	0	100	100	0	100	100	0,100,100	0	100	100
**Pattern B counts**	1	1	1	0	0	0	0	0	0	0

**Table 2:** Corner case scenarios. Scenarios A and B are generated by repeating hourly patterns A and B (Table 1) for 24 hours, midnight to midnight. Each scenario contains 1,440 one-minute epochs of count data.

	Scenario A	Scenario B
**Hourly pattern**	A	B
**Duration**	24 hours	24 hours
**Counts-per-hour**	4,000	3
**Counts total**	96,000	72

Scenarios A and B were evaluated using three implementations of the NCINW (i.e., SAS, ActiLife and RAP) to determine wear and nonwear time, as well as total counts accrued and total counts discarded as nonwear in each 24 hour simulation. This helped verify that the implementations were consistent with one another, and allowed us to more easily extend our analysis of the NHANES 2003–2004 data set next. Information concerning the specific configuration and setup of these software implementations and the necessary input data format is available in the supplement.

### NHANES 2003–2004 data

Next, we examined the prevalence of A and B type patterns in the NHANES 2003–2004 dataset, which includes 7,176 participants. The participants represent a diverse demographic of both youth and adults (6–85+ years in age), male and female. These differences do not affect NCINW’s behavior, which only considers the occurrence and quantity of counts in its classifications, and so we did not differentiate youth and adults in this analysis. The National Center for Health Statistics (NCHS) Ethics Review Board (ERB) obtained written consent from all participants and approved the NHANES 2003–2004 release of deidentified information to the public domain [29–31]. Stanford University's Panel on Human Subjects in Medical Research granted exemption from human participant’s review for our analysis of this data. Participants with less than 24 hours recorded, or whose equipment was not in calibration when returned, were excluded, leaving a total of 6,827 participants for our analysis.

Scenarios A and B were simulated to demonstrate NCINW’s most extreme behavior in a 24-hour day. Patterns like A and B may also occur naturally with similar, though less extreme levels of behavior. We use the number of counts in an hour, defined as T, to quantify these types of patterns. The specific ordering and magnitude of counts of A-type patterns consist of hours classified as nonwear with ≥ T counts for each hour, prior to classification (after classification, counts during nonwear are considered to be 0). B-type patterns represent hours classified as wear with ≤ T counts for each hour. Prevalence of A-type patterns was determined for T, evaluated on an interval from 10 to 500 counts per hour, and prevalence of B-type patterns was determined for T from 1 to 60 counts per hour. NCINW classification of wear and nonwear was done using the R *Accelerometry* package (see supplement for specific configuration). Only patterns that occurred during hourly clock intervals were considered (e.g. 1:00–1:59, but not 1:30–2:29); keeping with NCINW behavior.

Lastly, we explored how using the NCINW algorithm may impact the analysis of NHANES 2003–2004 using an approach similar to Troiano et al [4], as described in the introduction. Wear time and resulting counts were calculated using NCINW, and wear time classification was tallied for each 24-hour day. Days with ≥ 10 hours of wear time were deemed *valid* and considered for further. Participants with ≥ 1 and ≥ 4 valid days were tallied, and their average CPM calculated during valid day wear time, for each case. The number of minutes spent above ≥ 2,020 counts–the cutoff used by Troiano et al for moderate and vigorous physical activity in adults [4]–was calculated for valid days as well. We then showed how these metrics change when the standard, hourly, nonwear count allowance is restricted to a maximum threshold, T. To do this, A-type patterns, full hours initially classified as nonwear but which have T or more counts, were redesignated as wear time and allowed to contribute accordingly in order to survey the degree with which the specific corner cases described may impact the analysis. Setting T = 0 is equivalent to not applying the NCINW in any manner as all nonwear is reclassified as wear time (i.e. no data is excluded as nonwear). We evaluated T from 0 to 500, and showed each metric along with its difference from the NHANES 2003–2004 results found earlier, using NCINW’s default behavior.

## Results

Table 3 shows the nonwear and wear time classification results for Scenarios A and B. These results were consistent across NCISAS, ActiLife, and RAP. More than 99.9% of Scenario A was classified as nonwear, with 2 minutes of wear time and 200 counts classified as wear of the 24-hour, 96,000 counts input. This classification excludes the 95,800 counts and 23 hours and 58 minutes from analysis. Conversely, 100% of Scenario B was classified as wear; the three consecutive minutes of 1-counts per hour were enough to consider each hour as wear time for a total of 72 counts in the 24-hour period.

**Table 3:** Scenario results. Classification results for Scenarios A and B according to NCI’s nonwear classification algorithm. The results demonstrate worst case scenarios for misclassification of sedentary/light activity (Scenario A) and nonwear activity (Scenario B).

	Scenario A	Scenario B
**Before NCINW classification**		
Duration (HH:MM)	24:00	24:00
Counts-per-minute	66.67	0.05
Counts (total)	96,000	72
**After NCINW Classification**		
Duration wear (HH:MM)	00:02 (0.1%)	24:00 (100%)
Counts (total)	200	72
Duration excluded (HH:MM)	23:58 (99.9%)	00:00 (0%)
Counts excluded (total)	95,800	0

**Fig 1:**
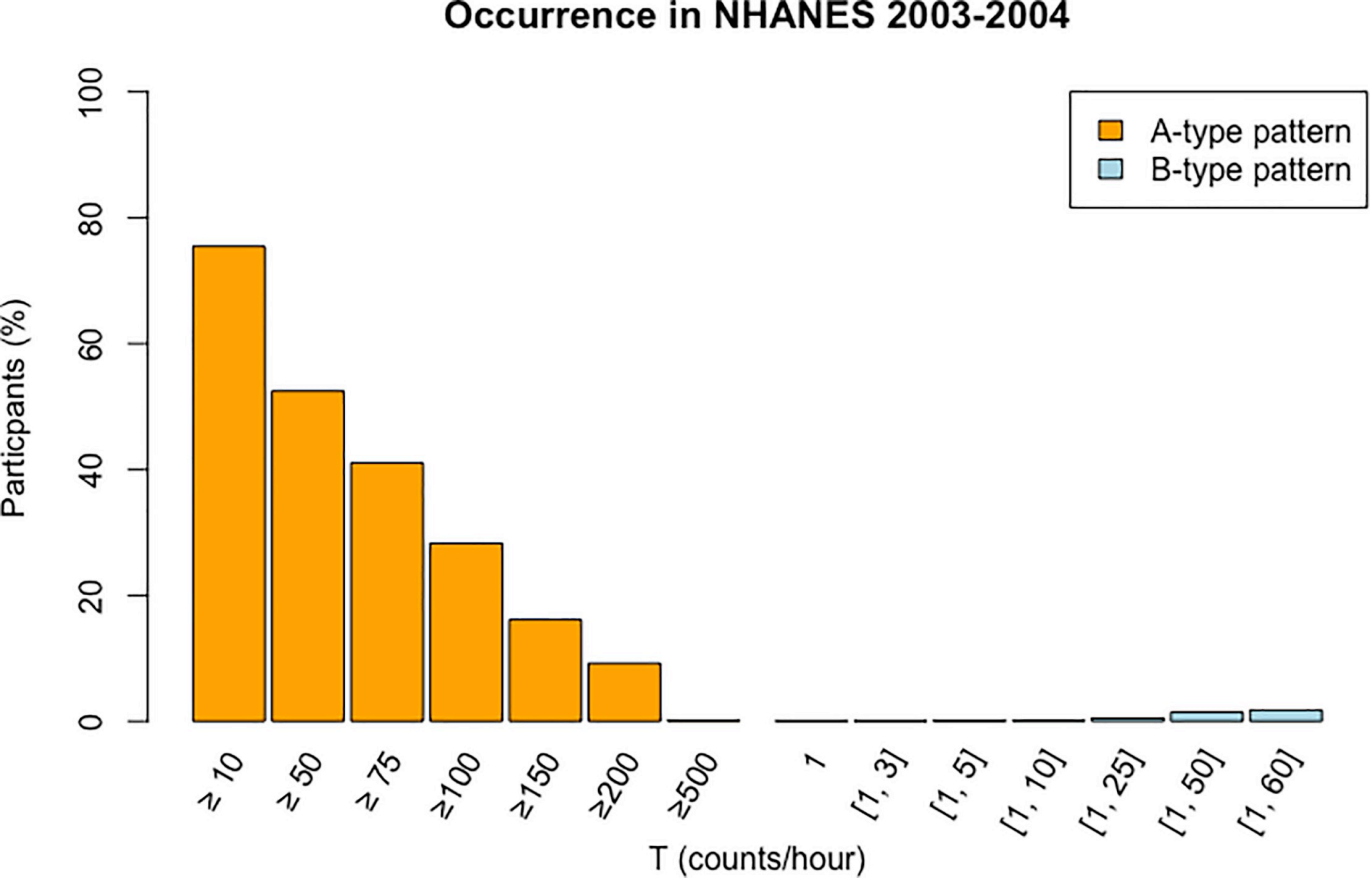
Prevalence in NHANES 2003–2004. The frequency of A-type and B-type patterns found in the NHANES 2003–2004 physical activity monitoring dataset is shown for different ranges of T. A-type patterns represent NCINW’s nonwear count allowance and consist of hours with ≥ T counts and classified as *nonwear* according to NCINW. B-type patterns comprise hours with between 1 and T counts that are classified as *wear*. Results are from examination of 6,827 participants.

Fig 1 shows the distribution of NHANES 2003–2004 participants examined (n = 6,827) having one or more A or B like hourly patterns, as determined using the RAP implementation of NCINW. There were 13 participants with one hour of 500 or more counts classified as nonwear by NCINW. There were five participants with an hour of five counts or fewer that occurred consecutively and were classified as wear by NCINW.

Table 4 shows the results of evaluating NHANES 2003–2004 according to different thresholds for reclassifying NCINW nonwear as wear. Including all data, T = 0, gives a higher number of valid days observed and a lower average CPM than the default. Increasing the counts-per-hour during nonwear threshold results in decreasing the number of participants with valid days while increasing their mean CPM. The number of participants with valid days converges to baseline for T ≥ 350. The average CPM is approximately 10 CPM fewer in participants with ≥ 4 valid days than it is for participants with ≥ 1 valid day, for T > 0. The average number of minutes, per day, with ≥ 2,020 counts was only slightly lower (less than one minute in all cases) for participants with ≥ 1 valid days compared to ≥ 4 valid days and varied by less than 1.3 minutes in each case for T ≥ 1.

**Table 4:** Analysis of NHANES 2003–2004 with varying nonwear allowance. Number of participants with ≥ 1 valid day and ≥ 4 valid days and the average counts-per-minute for each after applying NCI nonwear classification algorithm to the NHANES 2003–2004 PAM dataset and redesignating nonwear time with T or more counts-per-hour as wear time. A valid day is defined as ≥ 10 hours of wear time in a 24-hour day, beginning at midnight. Results are shown with difference and percent difference from baseline in parenthesis.

Nonwear inclusion criteria	1 or more valid days			4 or more valid days		
	Participants (n)	Counts-per-minute	Minutes-per-day at or above 2020 counts	Participants (n)	Counts-per-minute	Minutes-per-day at or above 2020 counts
**Default (none)**	6332	403.1	35.52	4873	392.2	34.46
T ≥ 500	6332 (0, 0.00%)	403.0 (-0.0, -0.00%)	35.52 (0.00, 0.00%)	4873 (0, 0.00%)	392.2 (-0.0, -0.00%)	34.46 (0.00, 0.00%)
T ≥ 400	6332 (0, 0.00%)	403.0 (-0.0, -0.01%)	35.52 (0.00, 0.00%)	4873 (0, 0.00%)	392.1 (-0.1, -0.01%)	34.46 (0.00, 0.00%)
T ≥ 350	6332 (0, 0.00%)	403.0 (-0.1, -0.02%)	35.52 (-0.00, -0.00%)	4873 (0, 0.00%)	392.1 (-0.1, -0.03%)	34.46 (-0.00, -0.00%)
T ≥ 300	6333 (1, 0.02%)	402.8 (-0.2, -0.06%)	35.52 (-0.01, -0.02%)	4875 (2, 0.04%)	392.0 (-0.2, -0.05%)	34.46 (-0.00, -0.01%)
T ≥ 250	6333 (1, 0.02%)	402.6 (-0.5, -0.12%)	35.51 (-0.02, -0.04%)	4876 (3, 0.06%)	391.7 (-0.5, -0.13%)	34.44 (-0.02, -0.05%)
T ≥ 200	6335 (3, 0.05%)	401.8 (-1.2, -0.30%)	35.47 (-0.06, -0.16%)	4882 (9, 0.18%)	391.1 (-1.1, -0.27%)	34.45 (-0.01, -0.04%)
T ≥ 150	6337 (5, 0.08%)	400.7 (-2.4, -0.59%)	35.43 (-0.10, -0.27%)	4886 (13, 0.27%)	390.0 (-2.2, -0.57%)	34.41 (-0.05, -0.15%)
T ≥ 100	6346 (14, 0.22%)	398.4 (-4.7, -1.17%)	35.35 (-0.18, -0.49%)	4902 (29, 0.59%)	387.6 (-4.6, -1.17%)	34.32 (-0.14, -0.40%)
T ≥ 90	6352 (20, 0.32%)	397.3 (-5.8, -1.44%)	35.31 (-0.22, -0.61%)	4915 (42, 0.86%)	386.9 (-5.3, -1.35%)	34.33 (-0.13, -0.38%)
T ≥ 80	6354 (22, 0.35%)	396.1 (-6.9, -1.73%)	35.22 (-0.30, -0.86%)	4928 (55, 1.12%)	386.9 (-5.3, -1.35%)	34.48 (0.02, 0.05%)
T ≥ 75	6356 (24, 0.38%)	395.7 (-7.4, -1.85%)	35.20 (-0.33, -0.92%)	4932 (59, 1.20%)	386.5 (-5.7, -1.46%)	34.48 (0.02, 0.05%)
T ≥ 70	6357 (25, 0.39%)	395.1 (-7.9, -1.98%)	35.18 (-0.34, -0.97%)	4938 (65, 1.33%)	386.0 (-6.2, -1.59%)	34.47 (0.01, 0.02%)
T ≥ 60	6362 (30, 0.47%)	393.9 (-9.1, -2.30%)	35.15 (-0.38, -1.07%)	4962 (89, 1.81%)	384.1 (-8.0, -2.07%)	34.34 (-0.12, -0.35%)
T ≥ 50	6372 (40, 0.63%)	392.7 (-10.4, -2.61%)	35.10 (-0.42, -1.20%)	4986 (113, 2.29%)	382.7 (-9.5, -2.45%)	34.27 (-0.19, -0.55%)
T ≥ 25	6393 (61, 0.96%)	388.5 (-14.6, -3.68%)	34.92 (-0.61, -1.72%)	5040 (167, 3.37%)	378.6 (-13.6, -3.53%)	34.11 (-0.35, -1.02%)
T ≥ 10	6415 (83, 1.30%)	383.7 (-19.3, -4.91%)	34.70 (-0.82, -2.33%)	5127 (254, 5.08%)	374.1 (-18.1, -4.73%)	33.92 (-0.54, -1.58%)
T ≥ 5	6433 (101, 1.58%)	380.0 (-23.0, -5.88%)	34.51 (-1.01, -2.89%)	5183 (310, 6.17%)	370.7 (-21.5, -5.62%)	33.75 (-0.71, -2.08%)
T ≥ 3	6440 (108, 1.69%)	378.1 (-25.0, -6.39%)	34.43 (-1.09, -3.12%)	5217 (344, 6.82%)	368.8 (-23.4, -6.15%)	33.68 (-0.78, -2.29%)
T ≥ 1	6457 (125, 1.95%)	374.5 (-28.5, -7.33%)	34.23 (-1.29, -3.70%)	5270 (397, 7.83%)	365.7 (-26.5, -6.98%)	33.54 (-0.92, -2.70%)
**T ≥ 0** **(all)**	6827 (495, 7.52%)	181.5 (-221.5, -75.80%)	26.53 (-9.00, -29.00%)	6823 (1950, 33.34%)	181.6 (-210.6, -73.41%)	26.54 (-7.92, -25.98%)

## Discussion

The NCINW is vulnerable to poor performance under two corner case conditions. Table 3 shows that, in Scenario A, NCINW will classify hours with 4,000 counts–patterns that are likely consistent with wear–as nonwear, whereas in Scenario B it will classify hours with only 3 counts–patterns that may be more consistent with nonwear–as wear. These simulated scenarios show the algorithm’s maximum and minimum count boundaries for wear and nonwear classification. They also highlight the potential problem of A-type and B-type patterns occurring in practice. Describing the algorithm’s allowance for nonwear as "1–2 min of counts between 0 and 100" in an hour of otherwise 0 activity counts [4] is correct, but perhaps not readily understood to also mean 1–40 min of counts between 1 and 4000, provided no minute has more than 100 counts and there are never more than two consecutive minutes with counts.

In examining the NHANES 2003–2004 dataset, with which the NCINW algorithm was provided in order to replicate its analysis, we found A-type patterns to be much more prevalent than B-type patterns. Fig 1 and the supplementary S3 Table show that 9.23% (n = 630) of the participants had at least one hour with 200 or more counts excluded as nonwear, and over half of the participants (52.45%, n = 3,581) had one or more hours with ≥ 50 counts excluded from analysis as nonwear. B-type patterns were less common with 1.79% of participants (n = 122) having one or more hours with 60 or fewer counts classified as wear, and only 0.19% of participants (n = 13) having one or more hours with 10 or less counts classified as wear. There were also four participants with one hour classified as wear, but containing only a single count; contrary to the algorithm’s definition. This was the first hour of the study for two participants, and the final hour of the study for the other two participants. Choi et al found inconsistent behavior when classifying activity during the last hour of a 24 hour day (11pm to midnight) using NCINW as well, and present a validated alternative which resolves these issues [15, 20].

The maximum number of counts any NCINW classified nonwear hour can have is 4,000 (Table 3). Setting the hourly nonwear allowance threshold to 4,001 counts is equivalent to NCINW’s default behavior. Table 4 shows that, as this threshold T was lowered and the number of A-type patterns reclassified as wear increased, the number of participants with valid days increased, and CPM and minutes-per-day at or above 2,020 counts, a proxy for bouts of physical activity, decreased. This may support Matthews et al. [28] deduction that, had the 1.5-hour discrepancy between survey-reported wear time and NCINW-based wear time (survey reported 1.5 hours more than NCINW) been included as wear, lower activity levels would have been observed. However, despite their prevalence, re-classifying A-type patterns as wear instead of nonwear did not meaningfully change the NHANES 2003–2004 summary results, compared to the default, for any T greater than 0. There was less than 5% difference for any metric with T > 10, and less than 1% difference with T > 100. The largest difference was a 7.83% increase in number of subjects with ≥ 4 valid days for T ≥ 1.

The most noticeable difference occurred when there was no effort to account for nonwear (i.e. T = 0) and all data were included (i.e., assuming no nonwear). In this case, mean CPM was 75.80% less for participants with ≥ 1 valid days, and 73.41% less for participants with ≥ 4 valid days when compared to NCINW’s standard nonwear criteria. The number of minutes-per-day with counts at or above 2,020 was -29.00% in participants with ≥ 1 valid days, and -25.98% in participants with ≥ 4 valid days. Interestingly, in the case of number-of-minutes spent above 2,020 counts for participants with ≥ 4 valid days, the maximum value (34.48 minutes) was reached at T = 75 and T = 80 counts per hour. It then dipped slightly before increasing again to the value of 34.46 minutes. The maximum number of counts allowed per minute during nonwear is capped at 100 counts by definition (Table 1), which means that additional, reclassified nonwear activity only contributes indirectly to this metric. Additional wear time helps bring some participants to the 10-hour wear time valid day criteria necessary for inclusion. There appears to be a pocket of participants here reflecting this aspect of the analysis.

There are several reasons the NHANES 2003–2004 analysis data set is protected from the potential misclassification observed in these corner case conditions. First, because these corner cases are less common, the large number of participants examined continuously for up to one week helps account for and generalize much of the results as intended. Second, the first cut-point for physical activity was set at 2,020 counts per-minute, well above the 100 count maximum allowed by NCINW to still be considered, and possibly excluded, as nonwear. Finally, the 10 hour, valid day criteria imposed on NHANES 2003–2004 bounds the examined data to a range of activity, most likely captured during daytime. Therefore, studies using smaller datasets, with shorter periods of observation, less restrictive inclusion criteria, or using lower count-per-minute cut-points, may be more susceptible to these corner case effects and need to exercise caution when using NCINW. In addition, studies of individual-level relationships between physical activity and/or sedentary behavior with other factors may be more sensitive to the effects of these corner cases than estimates of sample summary statistics. Further research is needed to clarify these points. We recommend using the NCINW with caution in the meantime.

## Conclusions

Our study showed specific, simulated conditions where NCI’s nonwear classification algorithm produces counterintuitive results. In Scenario A, hours with 4,000 counts each were classified as nonwear, while in Scenario B, hours with only 3 counts each were classified as wear. We examined the likelihood of finding similar patterns, A-type and B-type, within the NHANES 2003–2004 dataset and demonstrated how analysis of NHANES 2003–2004 is impacted when different levels of A-type patterns are reconsidered as wear. The uncertainty here is incorrectly classifying these times as nonwear when they may truly represent sedentary or sleeping behavior. The NCINW shows a bias towards classifying such activity as nonwear instead of low level, sedentary behavior. Despite these dangers and the prevalence of A-type patterns in NHANES 2003–2004, there was little change to the results for overall summary estimates of population levels of physical activity when accounting for these patterns. Still, the NCINW algorithm should be used with caution for smaller datasets, shorter periods of observation, and analysis with lower activity cut-points, and/or more lenient criteria for valid days of wear, or studies with different aims than NHANES 2003–2004, particularly including those that describe individual-level relationships between physical activity and an outcome, as opposed to summary statistics.

## Supporting information

S1 Text Software implementations of NCI’s Nonwear Classification Algorithm. (DOCX)

S1 Table Software implementations of NCI’s Nonwear Classification Algorithm. Software that implement the same wear/nonwear classification algorithm developed by the National Cancer Institute (NCI) to analyze the physical activity monitoring portion of the 2003–2004 National Health and Nutrition Examination Survey (NHANES 2003–2004).
(DOCX)

S2 Text Configuring R’s accelerometry package to mimic NCI’s Nonwear Classification Algorithm. (DOCX)

S2 Table NCI nonwear classification settings in R’s accelerometry package. The classification results for scenario A according to the R accelerometry package *accel*.*weartime* (RAP-2) method configured to match NCI’s SAS script (*nci = TRUE*) with default or unset values (parameter set 1) and non-default values (parameter set 2) set to match the original nonwear definition. The default parameters must be changed (parameter set 2) to mimic the NCISAS script.
(DOCX)

S3 Table Prevalence in NHANES 2003–2004. The number of NHANES 2003–2004 participants with one or more hours with ≥ T counts, but classified as *nonwear* according to NCINW, are shown in (A). The number of participants with one or more hours containing, inclusively between, 1 and T counts and classified as *wear*, are shown in (B). The percent of participants compared to the number evaluated (n = 6,827) is shown for each case.
(DOCX)
