# Supplementary material for: Potential corner case cautions regarding publicly available implementations of the National Cancer Institute’s nonwear/wear classification algorithm for accelerometer data
Source: PLoS One. 2018 Dec 31;13(12):e0210006. doi: 10.1371/journal.pone.0210006 (PMC6312247; doi:10.1371/journal.pone.0210006)
Supplement: S1 Text — (DOCX) [file pone.0210006.s001.docx]

**S1 Text. Software implementations of NCI’s Nonwear Classification Algorithm.**

NCINW was first implemented as part of a suite of SAS programs provided by NCI (NCISAS) [21], before being implemented by others. ActiGraph’s data analysis software ActiLife [22] and R’s accelerometry package (RAP) [15, 23] are two other programs which offer the same functionality as NCISAS (S1 Table). NCISAS was used to examine uniaxial accelerometer data collected from ActiGraph model 7164 uni-axial accelerometers (ActiGraph LLC, Pensacola, FL) set to measure vertical axis counts in 1-minute epochs.
