# Supplementary material for: Potential corner case cautions regarding publicly available implementations of the National Cancer Institute’s nonwear/wear classification algorithm for accelerometer data
Source: PLoS One. 2018 Dec 31;13(12):e0210006. doi: 10.1371/journal.pone.0210006 (PMC6312247; doi:10.1371/journal.pone.0210006)
Supplement: S1 Table — Software that implement the same wear/nonwear classification algorithm developed by the National Cancer Institute (NCI) to analyze the physical activity monitoring portion of the 2003–2004 National Health and Nutrition Examination Survey (NHANES 2003–2004). (DOCX) [file pone.0210006.s002.docx]

**S1 Table.**

| **Name of Software** | **Description** | **Granularity of Output** | **Software Sponsor or Maintainer (reference)** |
| --- | --- | --- | --- |
| NCISAS | NCI SAS program for classifying nonwear in NHANES 2003-2004. | 24 hr | NCI [21] |
| ActiLife | ActiLife Weartime Validation Tool, *Troiano 2007* selection | 24 hr | ActiGraph LLC [22] |
| RAP-1 | R accelerometry package (*accel.process*) | 24 hr | Van Domelen [23] |
| RAP-2 | R accelerometry package (*accel.weartime*) | 1 min | Van Domelen [23] |
