# Supplementary material for: Potential corner case cautions regarding publicly available implementations of the National Cancer Institute’s nonwear/wear classification algorithm for accelerometer data
Source: PLoS One. 2018 Dec 31;13(12):e0210006. doi: 10.1371/journal.pone.0210006 (PMC6312247; doi:10.1371/journal.pone.0210006)
Supplement: S2 Text — (DOCX) [file pone.0210006.s003.docx]

**S2 Text. Configuring R’s accelerometry package to mimic NCI’s Nonwear Classification Algorithm.**

ActiLife and RAP offer a variety of nonwear classification routines, and must be specifically configured the scenarios to mimic the format utilized by NCISAS. Specifically, we simulated Scenarios A and B using a tri-axial format that is recognized by these programs; placing the scenarios’ count values on the vertical axis and zero counts on the anteroposterior and mediolateral axes to mimic uniaxial behavior expected by NCISAS. Only the vertical axis counts are used for ActiLife and RAP’s implementation of NCI’s nonwear algorithm, however the tri-axial file format requires placeholders for vector magnitude counts, luminance, steps, and inclination which we assigned, respectively, as vertical counts, 0 lumens, 0 steps, and *standing*. We used ActiLife’s graphical user interface to select and configure their *Troiano (2007)* classification algorithm to match the original NCISAS parameters [22]. For RAP, we created and used an R script to process these files in the same way by invoking RAP’s *accel.process.tri* method with its NCI parameter set to true (i.e., *nci = TRUE*) (*RAP-1*). We also examined RAP’s *accel.weartime* method (*RAP-2*), which can be configured to output classified wear/nonwear time and counts at per minute granularity that, when aggregated back, mimic the daily summary levels provided by RAP-1. The S2 Table provides specific configuration instructions.

RAP-2’s aggregate results will differ from RAP-1’s, for Scenario A, if only the NCI implementation is set (*nci=TRUE*) and the other remaining parameters are left to take on their default. The S2 Table shows this, where *tol* represents the number of consecutive minutes allowed (inclusively between) 1 and the upper limit as set using *tol.upper*. The *days.distinct* parameter resets the counter allowance criteria at each new day when set to TRUE, and is discussed in detail by Choi et al [15]. The output for Scenario B is not affected by these differences. RAP-2, parameter set 2, matches the NCINW behavior, and was used in analyzing the NHANES 2003-2004 data set.
