# Supplementary material for: Potential corner case cautions regarding publicly available implementations of the National Cancer Institute’s nonwear/wear classification algorithm for accelerometer data
Source: PLoS One. 2018 Dec 31;13(12):e0210006. doi: 10.1371/journal.pone.0210006 (PMC6312247; doi:10.1371/journal.pone.0210006)
Supplement: S2 Table — The classification results for scenario A according to the R accelerometry package accel.weartime (RAP-2) method configured to match NCI’s SAS script (nci = TRUE) with default or unset values (parameter set 1) and non-default values (parameter set 2) set to match the original nonwear definition. The default parameters must be changed (parameter set 2) to mimic the NCISAS script. (DOCX) [file pone.0210006.s004.docx]

**S2 Table.**

|  | **Scenario A**  **(RAP-2, parameter set 1)** | **Scenario A**  **(RAP-2, parameter set 2)** |
| --- | --- | --- |
| **Ground truth** |  |  |
| Duration (hours) | 24 | 24 |
| Counts (per hour) | 4,000 | 4,000 |
| Counts (total) | 96,000 | 96,000 |
| **accel.weartime parameters** |  |  |
| Nci | TRUE | TRUE |
| Window | [unset,default=60] | [unset,default=60] |
| Tol | [unset,default=0] | 2 |
| tol.upper | [unset,default=99] | 100 |
| days.distinct | [unset,default=FALSE] | TRUE |
| **Classification results** |  |  |
| Portion nonwear/wear | **100%** **wear** | **99.9%** **nonwear** |
| Duration nonwear | 0 min | 23 hours 58 min |
| Duration wear | 24 hr | 0 hr 2 min |
| Counts (total) | 96,000 | 200 |
