# Supplementary material for: Potential corner case cautions regarding publicly available implementations of the National Cancer Institute’s nonwear/wear classification algorithm for accelerometer data
Source: PLoS One. 2018 Dec 31;13(12):e0210006. doi: 10.1371/journal.pone.0210006 (PMC6312247; doi:10.1371/journal.pone.0210006)
Supplement: S3 Table — The number of NHANES 2003–2004 participants with one or more hours with ≥ T counts, but classified as nonwear according to NCINW, are shown in (A). The number of participants with one or more hours containing, inclusively between, 1 and T counts and classified as wear, are shown in (B). The percent of participants compared to the number evaluated (n = 6,827) is shown for each case. (DOCX) [file pone.0210006.s005.docx]

**S3 Table.**

1. Occurrence of pattern A-type hours in NHANES 2003-2004.

| T (counts per hour) | ≥ 10 | ≥ 50 | ≥ 75 | ≥ 100 | ≥ 150 | ≥ 200 | ≥ 500 |
| --- | --- | --- | --- | --- | --- | --- | --- |
| Participants (n) | 5150 (75.44%) | 3581 (52.45%) | 2805 (41.09%) | 1930 (28.27%) | 1104 (16.17%) | 630 (9.23%) | 13 (0.19%) |

1. Occurrence of pattern B-type hours in NHANES 2003-2004.

| T (counts per hour) | [1] | [1, 3] | [1, 5] | [1, 10] | [1, 25] | [1, 50] | [1, 60] |
| --- | --- | --- | --- | --- | --- | --- | --- |
| Participants (n) | 4  (0.06%) | 5  (0.07%) | 8 (0.12%) | 13 (0.19%) | 35 (0.51%) | 99 (1.45%) | 122 (1.79%) |
